# Joint modelling of time-to-event and multivariate longitudinal outcomes: recent developments and issues

**DOI:** 10.1186/s12874-016-0212-5

**Published:** 2016-09-07

**Authors:** Graeme L. Hickey, Pete Philipson, Andrea Jorgensen, Ruwanthi Kolamunnage-Dona

**Affiliations:** 1 Department of Biostatistics, University of Liverpool, Waterhouse Building, 1-5 Brownlow Street, Liverpool, L69 3GL UK; 2 Department of Mathematics, Physics and Electrical Engineering, Northumbria University, Ellison Place, Newcastle upon Tyne, NE1 8ST UK

**Keywords:** Joint models, Multivariate data, Longitudinal data, Time-to-event data, Software

## Abstract

**Background:**

Available methods for the joint modelling of longitudinal and time-to-event outcomes have typically only allowed for a single longitudinal outcome and a solitary event time. In practice, clinical studies are likely to record multiple longitudinal outcomes. Incorporating all sources of data will improve the predictive capability of any model and lead to more informative inferences for the purpose of medical decision-making.

**Methods:**

We reviewed current methodologies of joint modelling for time-to-event data and multivariate longitudinal data including the distributional and modelling assumptions, the association structures, estimation approaches, software tools for implementation and clinical applications of the methodologies.

**Results:**

We found that a large number of different models have recently been proposed. Most considered jointly modelling linear mixed models with proportional hazard models, with correlation between multiple longitudinal outcomes accounted for through multivariate normally distributed random effects. So-called current value and random effects parameterisations are commonly used to link the models. Despite developments, software is still lacking, which has translated into limited uptake by medical researchers.

**Conclusion:**

Although, in an era of personalized medicine, the value of multivariate joint modelling has been established, researchers are currently limited in their ability to fit these models routinely. We make a series of recommendations for future research needs.

**Electronic supplementary material:**

The online version of this article (doi:10.1186/s12874-016-0212-5) contains supplementary material, which is available to authorized users.

## Background

In many clinical studies, subjects are followed-up repeatedly and response data collected, for example a biomarker. The time to an event is also usually of interest, which might be explicit, e.g. death, or implicit, e.g. dropout. The longitudinal data may be censored by this time-to-event outcome. Modelling the longitudinal and event-time outcomes separately, for example using linear mixed models [1] or Cox regression models [2], can therefore be inefficient, and can lead to biased effect size estimates if the two outcome processes are correlated [3].

Research into joint modelling of longitudinal and time-to-event data has received considerable attention during the past two decades [3–7]. The motivation behind this active field of research has stemmed from three broad scientific objectives:

1. Improving inference for a repeated measurement outcome subject to an informative dropout mechanism that is not of direct interest [8].
2. Improving inference for a time-to-event outcome, whilst taking account of an intermittently and error-prone measured endogenous time-dependent variable [5].
3. Studying the relationship between the two correlated processes [6].

Depending on the objective, previous approaches to joint modelling have included fitting time-dependent Cox models [2], and two-stage models [9, 10]. In each case, joint modelling can lead to improvements in the efficiency of statistical inferences and reduces bias [11], which can yield substantial benefits when designing trials. Joint models can also be used to improve prediction [12], or to determine whether a longitudinal process is a surrogate for a time-to-event process [10]. The literature is extensive, with comprehensive reviews given by Hogan and Laird [13], Tsiatis and Davidian [14], and Gould et al. [15].

Previous research has mostly concentrated on the joint modelling of a single longitudinal outcome and a single time-to-event outcome; herein referred to as *univariate joint modelling*. Commensurate with this methodological research has been an increase in wide-ranging clinical application [16–21] and accessibility to analysis tools using mainstream statistical software packages [22–28]. In practice, however, the data collected will often be more complex, featuring multiple longitudinal outcomes and possibly multiple, recurrent or competing event times. As an example, Rizopoulos and Ghosh [29] described data on 407 patients with chronic kidney disease who underwent a renal transplantation. Each patient had 3 separate biomarker measurements repeatedly recorded: glomerular filtration rate (GFR), blood haematocrit level, and proteinuria. Each of these can be considered as markers of renal function, with the clinical interest being the time to graft failure. Harnessing all available information in a single model is advantageous and should lead to improved model predictions. This therefore makes multivariate joint modelling an attractive tool in an era of personalized medicine, as physicians can gain a better understanding of the underlying disease dynamics and ultimately choose the most optimal treatment for a patient at a particular follow-up time point. Notwithstanding the increased flexibility and better predictive capabilities, the extension of the classical univariate joint modelling framework to a multivariate setting introduces a number of technical and computational challenges.

A number of factors, many still faced by the univariate joint modelling framework, precludes integration of this more sophisticated modelling framework into routine clinical research practice [15]. The aim of this paper is to provide an overview of recent methodological developments and applications of joint models for time-to-event data and multivariate longitudinal data (MVJMs).

## Methods

A MVJM is comprised of two submodels: (1) a multivariate longitudinal data model, and (2) a time-to-event data model.

Let *Y*_*ik*_(*t*_*ijk*_) denote the *j*-th observed value of the *k*-th longitudinal outcome for subject *i*, measured at time *t*_*ijk*_, for *i* = 1, …, *N*; *k* = 1, …, *K*, and *j* = 1, …, *n*_*ik*_. There are a plethora of modelling approaches for multivariate longitudinal data [30]. A generalized linear mixed model (GLMM) is a common approach, where measurements for different outcomes can be recorded at different times between patients and outcomes, and is given by:

1 $$ {h}_k\left\{E\left[{Y}_{ik}\left({t}_{ijk}\right)\right]\right\} = {\mu}_{ik}\left({t}_{ijk}\right), $$

where *h*_*k*_(⋅) denotes a known one-to-one link function for the *k*-th outcome, and *μ*_*ik*_(⋅) is the linear predictor:

2 $$ {\mu}_{ik}\left({t}_{ijk}\right)={X}_{ik}^{(1)}\left({t}_{ijk}\right){\beta}_k^{(1)} + {Z}_{ik}\left({t}_{ijk}\right){b}_{ik}, $$

where *X*_*ik*_^(1)^(*t*_*ijk*_) and *Z*_*ik*_(*t*_*ijk*_) are row-vectors of (possibly time-varying) covariates for subject *i* associated with fixed and random effects respectively, which can vary by outcome; *β*_*k*_^(1)^ is a vector of fixed effects parameters for the *k*-th outcome; and *b*_*ik*_ is a vector of subject-specific random effects for the *k*-th outcome. We denote the stacked vector of subject-specific random effects for all *K* outcomes by *b*_*i*_ = (*b*_*i*1_^*T*^, *b*_*i*2_^*T*^, …, *b*_*iK*_^*T*^)^*T*^.

Cox’s proportional hazards (PH) semiparametric model [2] has been a common choice for the time-to-event submodel when modelling continuous event times. For a single failure-time per subject, the hazard function for subject *i* at time *t* is given by

3 $$ {\lambda}_i(t) = {\lambda}_0(t) \exp \left\{{X}_i^{(2)}(t){\beta}^{(2)}+{W}_i(t)\right\}, $$

where *X*_*i*_^(2)^(*t*) is a (possibly time-varying) row-vector of external covariates; *λ*_0_(*t*) denotes the baseline hazard function; *β*^(2)^ is a vector of fixed effects parameters; and *W*_*i*_(*t*) is a latent process that captures the association structure between the measurement and event processes. When the PH assumption is not met, alternative modelling frameworks can be exploited, such as accelerated failure time models, which can be written directly in terms of the survival function, *S*_*i*_(*t*), as

$$ {S}_i(t) = {S}_0\left( \exp \left\{{X}_i^{(2)}(t){\beta}^{(2)}+{W}_i(t)\right\}t\right), $$

where *S*_0_(⋅) is the baseline survival function that depends on the parametric family used for modelling, and all other parameters are defined as per the PH model (3). Discrete event times can also be jointly modelled with longitudinal data, particularly for selection models, which is applicable to situations of interval-censored continuous event times and predefined measurement schedules. Models here might include logistic or probit regression models [31]. We can generally represent the discrete distribution function as a function of the latent association term, namely *F*(*X*_*i*_^(2)^(*t*_*j*_)*β*^(2)^ + *W*_*i*_(*t*_*j*_)).

**Fig. 1:**
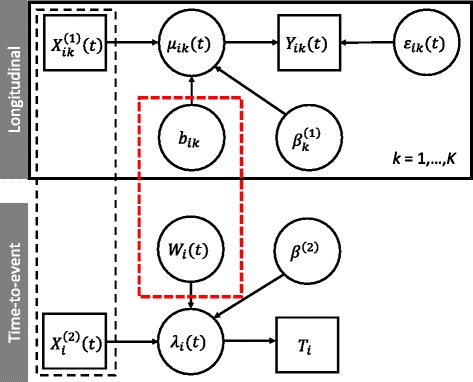
Graphical representation of a joint model of a time-to-event submodel and *K*-multivariate longitudinal outcomes submodel. Square boxes denote observed data; circles denote unobserved (including random) terms. The black-dashed box indicates that covariates can be shared between both submodels. The red-dashed box indicates that the process *W*_*i*_(*t*) and the random effects, *b*_*i*_, are correlated, which gives rise to the joint model. *T*_*i*_ is the failure time, which may or may not be observed, in which case a censoring time is observed. All other notation is defined as above

In principle, each submodel can be fitted separately. However, this can result in biased estimates and a loss of efficiency when the processes are correlated. When *b*_*ik*_ and *W*_*i*_(*t*) are jointly modelled, it leads to the so-called shared random effects joint model. A graphical representation of this model is shown in Fig. 1. Joint models might also fall under the umbrellas of pattern-mixture models or selection models depending on the factorization of the joint distribution of event time and longitudinal data [31, 32]. Furthermore, modelling approaches might also fall under the umbrellas of joint latent class models [33], semiparametric [34], and fully parametric models [35]. We note also that the topic of missing values in longitudinal data analyses has its own literature [36]. In the MVJM literature, focus has mainly been towards shared random effects models. We review the different submodels, including distributional assumptions and correlation structures, latent association structures, estimation techniques, software implementations, and give clinical examples of application.

## Results

### Longitudinal data submodel

The choice of model for the longitudinal outcome data will depend on the type of data measured (continuous, ordinal, discrete). Although in the development and application of MVJMs they are often restricted to the simple case of continuous outcomes only [17, 19–21, 37–53], it is conceivable that multiple outcomes might be a mixture of different outcome types. For example, in Rizopoulos and Ghosh [29], GFR and haematocrit were both continuous measures, whereas proteinuria was recorded as a binary outcome. More recent modelling approaches developed have incorporated combinations of different outcome types [4, 18, 29, 54–65]. Other models have considered multivariate binary [66], ordinal [67, 68], and continuous [0,1]-bounded data [44, 45, 47, 53]. Thiébaut et al. [41] and Guedj et al. [52] both proposed a model for multiple continuous outcomes, but also allowed for left-censoring, which is a pertinent issue with biomarker measurements as they can fall below the minimum detection level.

#### Model

For continuous longitudinal outcomes, the Laird and Ware [1] linear mixed model (LMM) with independent and identically normally distributed within-subject measurement errors is ubiquitous. However, this distributional assumption can be sensitive to outlier observations and heavy-tailed data, which has prompted robust modelling considerations in the univariate framework [69]. Despite this, it has translated into only limited model innovation in the MVJM framework, with Tang and Tang [49] considering using the multivariate skew-normal distribution. The robustness of model estimates to misspecification of errors, error structures, and magnitude of errors, has been examined through several simulation studies [40, 43, 49, 50]. Dantan et al. [44] and Proust-Lima et al. [45, 57] considered a model for [0, 1]-bounded continuous data using the Beta transformation link function, as it is parsimonious and offers very flexible shapes. This, however, might require a preliminary rescaling of the markers. Liu and Li [47] and Hatfield et al. [53] also considered data on the [0, 1] interval, but instead used zero-one inflated and ‘zero-augmented’ beta regression models, respectively. Guedj et al. [52] proposed a completely different approach by modelling multivariate continuous biomarkers using a mechanistic model based on a system of nonlinear ordinary differential equations.

For binary and count data, the standard model is the GLMM. This model has been regularly used in the MVJM framework [4, 18, 29, 54–56, 58, 60–63, 66, 70]. Moreover, this can be extended to multivariate data of different outcome types (e.g. a combination of continuous and binary measures) with correlation induced by modelling the random effects together. Huang et al. [66] considered a logistic regression model for binary outcome data, with a logistic regression model for the unobserved latent variable and a linear pairwise odds-ratio model for the association between marginal probabilities. Models for ordinal data have been considered, including the proportional odds model [18, 54, 55, 64, 67], cumulative probit model [57], partial proportional odds model [68], and the continuation ratio mixed model [56, 59].

#### Random effects distributions

In the univariate joint model framework, it has been reported that inferences are generally robust to misspecification of random effects [71, 72]. However, in the MVJM framework the dimension of these random effects might be greater, potentially amplifying the impact of misspecification on parameter estimates and standard errors. In general, the ubiquitous normality assumption might be too restrictive to capture individual-level variation [49]. Nonetheless, multivariate normal distributions are the standard modelling choice for random effects in the longitudinal submodel. Several simulation studies have generated data under misspecified random effects [43, 49, 60, 68]. Pantazis and Touloumi [43] explored misspecification by fitting their proposed model [40] to data simulated under a range of heavy tailed, skewed, and mixture distributions. They concluded that fixed effect parameter estimates were quite robust to misspecification with the exception of those in the time-to-event submodel, but standard errors may be underestimated for heavily skewed distributions. These findings were in agreement with those found by Li et al. [68]. Xu et al. [58] explored the sensitivity of the parameter estimates to a multivariate *t*-distribution for the random effects. Tang and Tang [60] investigated the effect of misspecification on their semiparametric model by simulating data with random effects drawn independently from uniform and relocated Gamma distributions. Song et al. [38] simulated random effects from a bimodal normal mixture distribution to confirm the robustness of the semiparametric estimator; whereas Rizopoulos and Ghosh [29] simulated random effects from a three-component normal mixture model to confirm the robustness of the Dirichlet process prior formulation.

To avoid misspecification, it can be advantageous to semiparametrically model the random effects. In the Bayesian paradigm, extended from the univariate framework [73], a Dirichlet process has been used to specify the random effects prior distribution [29, 49, 60]. The subject-specific random effects were assumed to be independently and identically distributed according to some unknown density function, which is modelled by the Dirichlet prior [74]. Song et al. [38] treated the random effects as nuisance parameters, for which a set of estimating equations were deduced based on a derived sufficient statistic. Li et al. [68] also proposed a method whereby the distributions of the assumed zero-mean random effects could be left completely unspecified and estimated entirely non-parametrically by exploiting the vertex-exchange method.

Discrete random effects confer an advantage in model estimation by replacing (possibly high-dimensional) numerical integration by summations that are more manageable. Bartolucci and Farcomeni [61] introduced two discrete random effects: one that follows a single first-order latent Markov chain distribution, and a second time-constant latent variable to account for unobserved heterogeneity. Huang et al. [66] also used independent discrete random effects to account for association between events and longitudinal outcomes.

#### Correlation structure

Multidimensional random effects are usually treated as correlated and modelled with a multivariate normal distribution with unstructured covariance matrix; however, independence and other structures have been considered. Ibrahim et al. [19] considered an unstructured correlation between the random effects, but also explored using a more parsimonious (and computationally faster) diagonal covariance matrix. Proust-Lima et al. [45, 57] included subject-class-level specific random effects in the latent variable model, and subject-outcome-level specific random intercepts in the longitudinal submodel. The subject-outcome random-intercepts were distributed normally with outcome-specific variance. The subject-class random effects, however, were distributed multivariate normally with latent-class specific mean and an unstructured covariance matrix multiplied by a (latent) class-specific proportionality parameter. Musoro et al. [17] considered two types of random effects in the multivariate longitudinal submodel: the standard subject-specific random effects modelled by a multivariate normal distribution, and basis-outcome-specific random effects for the thin-plate spline used to model time, modelled according to independent normal distributions with basis-outcome specific variances. Li et al. [68] and Choi et al. [65] factorised the random effects as *b*_*ik*_ = Γ_*k*_*b*_*i*_, which reduces the dimension of random effects. For large *K*, Hatfield et al. [53] also discussed alternative simpler correlation structures for the random effects.

Although correlation between the multiple longitudinal outcomes can be modelled through the subject-specific random effects, it can alternatively be modelled through correlated error terms [19, 20, 38, 39, 42, 49, 60, 70]. Using the notation from (2) assuming a multivariate LMM with normal errors and multiple outcomes recorded according to a single measurement schedule, we have

$$ {Y}_{ik}\left({t}_{ij}\right) = {X}_{ik}^{(1)}\left({t}_{ij}\right){\beta}_k^{(1)} + {Z}_{ik}\left({t}_{ij}\right){b}_{ik} + {\varepsilon}_{ijk}. $$

Let also *ε*_*ij* ⋅_ = (*ε*_*ij*1_^*T*^, …, *ε*_*ijK*_^*T*^)^*T*^ denote the vector of errors for the *k*-th outcome. Xu and Zeger [58] considered the approach of correlated subject-specific random effects; namely

$$ {\varepsilon}_{ijk}\sim N\left(0,{\sigma}_k^2\right),\kern0.5em \mathrm{and}\kern0.5em {b}_i\sim {N}_v\left(0,\Psi \right), $$

where *σ*_*k*_^2^ is an outcome-specific variance term, and Ψ is a (*v* ﻿×﻿﻿﻿ *v*)*-*covariance matrix capturing the correlation between markers and repeated measures. Chi and Ibrahim [42] on the other hand considered the approach of correlated error terms; namely:

$$ {\varepsilon}_{ij}. \sim {N}_K\left(0,\ \Sigma \right),\kern0.5em \mathrm{and}\kern0.5em {b}_{ik}\sim {N}_{v_k}\left(0,\ {\Psi}_k\right), $$

where Σ is a covariance matrix that captures the association between longitudinal outcomes measured at the same time, and Ψ_*k*_ is a covariance matrix that captures the association between the random effects for the *k*-th outcome (with the vector *b*_*ik*_ of length *v*_*k*_). Chi and Ibrahim noted that their model allows for dependence between repeated measures and correlation between longitudinal outcomes to be considered separately, whereas in the Xu and Zeger model the different correlation types are conflated. Notwithstanding the inferential benefit, the latter model requires additional covariance parameters be estimated, which increases the computational challenge. Interestingly, Baghfalaki et al. [64] replaced the random effects term completely independent of outcome *k*, which whilst allowing for correlation between outcomes, in general will not be biologically plausible as subject-specific deviations will be on different scales for disparate longitudinal outcomes.

An alternative approach to model correlation between multiple longitudinal outcomes is to introduce a common latent variable between the models. Ibrahim et al. [19] considered the model

$$ {Y}_{ik}\left({t}_{ij}\right) = {\beta}_{0k} + {\beta}_{1k}{\mu}_i\left({t}_{ij}\right) + {\varepsilon}_{ijk}, $$

where *μ*_*i*_(*t*_*ij*_) is the true unobserved latent variable at time *t*_*ij*_. For example, if *K* different immunological measurements were recorded from a blood sample at time *t*_*ij*_, then we might let *μ*_*i*_(*t*_*ij*_) denote the ‘true antibody level’, which we cannot observe, but which we can infer from the multivariate outcome. The model is completed by specifying the latent variable model, e.g. as in (2), and a distribution for the error terms. For example, Ibrahim et al. considered *ε*_*ij*_. ∼ *N*_*K*_(0, Σ), and Luo [55], *ε*_*ijk*_ ∼ *N*(0, *σ*_*k*_^2^). Guedj et al. [52] intrinsically accounted for correlation between multiple biomarkers through the system of dependent ODE equations that models the biological system.

Within-subject autocorrelation structures, e.g. Henderson et al. [6], are not routinely considered; although Wang et al. [67] introduced a Gaussian process model for the underlying latent variable with a power function of time modelling the correlation. Proust-Lima et al. [57] considered the inclusion of a flexible zero-mean Gaussian autocorrelated process that admits Brownian motion and autoregressive processes as special cases. Ibrahim et al. [19], Pantazis et al. [40], and Hatfield et al. [53] noted their models could be extended to autocorrelation, but at the expense of added computational burden.

In some cases, where multivariate longitudinal data has been collected, the correlation has been ignored in order to allow simpler univariate joint models to be applied. For example, Battes et al. [21] used an ad hoc approach of either summing or multiplying the three repeated continuous measures (standardized according to clinical upper reference limits of the biomarker assays), and then applying standard univariate joint models.

### Time-to-event data submodel

Commonly, event times are measured continuously. If the subject does not experience the event of interest during the study observation period, then they are right-censored at the last known follow-up time. In some cases, where there is delayed entry to the study, it is necessary to account for left truncation [45, 57, 62]. In the MVJM framework, discrete event times have also been considered, [46, 50, 51, 61, 66]. Bartolucci and Farcomeni [61] noted that their estimation method could be extended to include continuous right-censored event times at a cost of loss of information and efficiency. Dantan et al. [44] also considered discrete time, but the model applied was relevant to continuous time also. These models do not permit premature non-informative censoring before the end of the study. However, Albert and Shih [46] suggested that if subjects are censored before the end of the study, then a separate precursory model could be used to impute the event times prior to application of the joint model methodology.

In practice, clinicians might not only be interested in multivariate longitudinal outcomes, but also multivariate time-to-event data. For example, Chi and Ibrahim [42] were interested in assessing whether different quality of life measures were prognostic and predictive of breast cancer progression in a drug randomised controlled trial (RCT). In addition to the multivariate longitudinal outcomes, the study monitored patients concerning two event times: overall survival and disease-free survival. Tang et al. [60], Tang and Tang [49], and Zhu et al. [70] each proposed *multiple events* joint models, motivated by the same dataset as analysed by Chi and Ibrahim [42]. Musoro et al. [17] considered a case of *multiple recurrent events*, where each patient could become repeatedly infected with one of 9 different infections. Huang et al. [66] analysed data from a complex prevention trial, with an interest on whether different interventions were associated with times to initiation of alcohol use and tobacco use. Competing risks data have recently been considered in the context MVJMs also [56, 57, 59, 68].

#### Model

The Cox PH model is an attractive choice as no distributional assumptions are required on the time-to-event data. In some settings, the unspecified baseline hazards function has been replaced by either a piecewise constant step-function for some preselected knots [18, 19, 29, 39, 47, 49, 54, 57, 59, 60, 70], or modelled using spline functions, including B-splines [4, 56], M- (and I-) splines [45, 57], and restricted cubic splines [62]. The position of knots is usually decided in advance, for example by taking quantiles (e.g. [59]), though advice is generally lacking. As an added degree of flexibility, Proust-Lima et al. [45, 57] consider class-specific (and cause-specific where applicable) baseline hazards that can be either stratified by class *m* = 1, …, *M*, i.e. *λ*_0*m*_(*t*), or proportional by class, i.e. exp(*β*_*m*_)*λ*_0_(*t*), for some parameters *β*_*m*_.

Parametric models considered in the MVJM framework include the Weibull [20, 45, 52, 53, 55, 57, 62, 64, 65], exponential [20, 52, 62, 65], log-normal [40, 41, 43, 55, 64], log-logistic [55], and Gompertz [57, 62]. Hu et al. [48] used a Weibull model for imputing composite event times, but used a conditional multinomial logistic regression model to then impute the cause type. The software package by Crowther [62] also allows for the Royston-Parmar model [75] to be fitted, which is a flexible parametric model that models the log-cumulative hazard using restricted cubic splines. For models involving multivariate event time data, standard models were applied; notably, for competing risks data the cause-specific hazards model was applied [76]. Chi and Ibrahim [42] developed a novel multivariate survival model that accommodates both zero and non-zero cure fractions, and which has a PH structure conditionally and marginally under certain settings. Hu et al. [48] developed an imputation approach that first imputed a composite event time, and then imputed a cause type using a conditional multinomial regression model.

The pattern-mixture model approach by Fieuws et al. [63] used the Kaplan-Meier estimator to model the failure time, which they described as prior probabilities, which were used in an elegant Bayes rule to calculate the conditional probability of failure. Models considering discrete time data have utilised a number of discrete probability models, including the probit model [46], logistic model [50], discrete time log-linear hazard models [66], truncated geometric distribution [50, 51, 61], and discrete proportional hazards model [50].

#### Frailty

Random effects are also included in some time-to-event submodels, to account for correlation between different or repeated events, where they are referred to as frailty effects when multiplicative on the hazard. Chi and Ibrahim [42] proposed a power stable law distribution to account for correlation between multiple event times. Lin et al. [37] and Choi et al. [65] respectively included gamma and log-normally distributed frailty terms to allow for subject-level variability.

### Association structures

Fundamental to the joint modelling framework is the association structure between the longitudinal submodel and the time-to-event submodel. Rationale for selecting this association structure has received relatively little attention. McCrink et al. [32] state that the choice of association structure should reflect the study focus, namely whether it is with respect to the time-to-event or longitudinal submodel (or both). For a discussion of different association structures for univariate joint models, see Rizopoulos [4]. We consider below different representations for *W*_*i*_(*t*) applied in the MVJM framework (Table 1). MVJMs that fall outside of the ubiquitous shared random effects framework cannot be reduced to simply specifying *W*_*i*_(*t*), and so we describe these models separately. In some cases, one might want to use different association structures for different longitudinal outcomes. This is a greatly overlooked modelling issue, which to the best of our knowledge, only Andrinopoulou et al. [56] and Crowther [62] have considered.

**Table 1:** Some association structures for joint models of time-to-event and multivariate longitudinal data

Parameterization	Latent association	Studies^i^
A1: Current value (linear predictor)	${W}_i(t)={\displaystyle {\sum}_{k=1}^K{\alpha}_k{\mu}_{ik}(t)}$	[17, 19, 21, 29, 37, 39, 42, 48, 49, 52, 56, 58, 60, 62, 67, 70]
A2: Current value (expected value)^a^	${W}_i(t)={\displaystyle {\sum}_{k=1}^K{\alpha}_k{h}_k^{-1}\left({\mu}_{ik}\right(t\left)\right)}$	[62]
A3: Interaction^b^	${W}_i(t)={\displaystyle {\sum}_{k=1}^K\left\{{\alpha}_k{\mu}_{ik}(t)+{\sum}_{l=1}^p{x}_{il}^{(2)}{\mu}_{ik}(t){\gamma}_{kl}\right\}}$	[37]
A4: Lagged time^c^	${W}_i(t)={\displaystyle {\sum}_{k=1}^K{\alpha}_k{\mu}_{ik}\left(t-c\right)}$	[46]
A5: General vector function^d^	${W}_i(t)={a}^T\psi \left(t,,,{b}_i\right)$	[38]
A6: Time-dependent slopes^e^	${W}_i(t)={\displaystyle {\sum}_{k=1}^K\left\{{\mu}_{ik}(t)+{\displaystyle {\sum}_{v=1}^V{\alpha}_{vk}\frac{d^v}{d{t}^v}{\mu}_{ik}(t)}\right\}}$	[29, 56]
A7: Cumulative effects	${W}_i(t)={\displaystyle {\sum}_{k=1}^K{\alpha}_k{\displaystyle {\int}_0^t}{\mu}_{ik}(s)ds}$	[56]
A8: Random effects^f^	${W}_i(t)={\displaystyle {\sum}_{k=1}^K{\alpha}_k^T{b}_{ik}}$	[18, 29, 53–55, 59, 62, 64, 65, 68]
A9: Generalised random effects + fixed effects^g^	${W}_i(t)={\displaystyle {\sum}_{k=1}^K{\alpha}_k^Tr\left({b}_{ik} + {\tilde {\beta}}_k^{(1)}\right)}$	[29, 50, 51, 59, 62]
A10: Correlated random effects^h^	${W}_i(t)={\theta}_i{\textstyle}\mathrm{with}{\textstyle }{\left({b}_i^T,,,{\theta}_i\right)}^T\sim {\mathrm{F}}_{\mathrm{a}}$	[29]

Notation: *μ*_*ik*_(*t*) denotes the linear predictor term of the longitudinal submodel for subject *i* and outcome *k*; *α*_*k*_ denotes the association parameter for the *k*-th outcome

^a^ *h*_*k*_(⋅) is the link function for the *k*-th outcome

^b^ *x*_*il*_^(2)^ denotes the *l*-th baseline covariates for subject *i* (*l* = 1, …, *p*) with corresponding coefficient parameters *γ*_*kl*_ for each outcome *k*. In practice, some *γ*_*kl*_ coefficients will be set to zero

^c^ *c* is a lag time (with *c* = 0 returning the current value parameterization). In Albert and Shih [46], time was modelled discretely and a selection model adopted, such that *W*_*i*_(*t*_*j*_) = ∑_*k* = 1_^*K*^ *α*_*k*_ *μ*_*ik*_(*t*_*j* − 1_)

^d^α is a vector of association parameters and *ψ*(*t*, *b*_*i*_) is a vector of time and random effects. It is assumed that *ψ*(*t*, *b*_*i*_) can be decomposed as *ψ*(*t*, *b*_*i*_) = *ψ*(*t*)*b*_*i*_. This general parameterization admits the current value parameterization as a special case, and leads to a number of extensions including interactions with time. In cases where *ψ*(*t*, *b*_*i*_) does not factorise, the authors propose using an approximation method

^e^ *α*_*vk*_ denote additional association parameters for the *ν*-th derivative (with respect to time) for the *k*-th longitudinal outcome mean trajectory function

^f^ *α*_*k*_ denotes a vector of association parameters of same dimension as the number of random effects for each outcome. In practice, some elements of *α*_*k*_ might be forced to zero, e.g. if only random intercepts were used to link the model

^g^as per the random effects parameterization *α*_*k*_ denotes a vector of association parameters of same dimension as the number of random effects for each outcome. ${\tilde {\beta}}_k^{(1)}$ denotes the subset of coefficient parameters from *β*_*k*_^(1)^ that correspond to the random effect terms, and *r*(⋅) denotes a vector function. If *r*(⋅) is the identify function, then the standard random + fixed effects parameterization is returned

^h^ *F*_*α*_ denotes a multivariate density function with parameters *α* to model correlation

^i^Rizopoulos [4] describes a general MVJM and notes that, in principle, the general association structures that are used in the R JM package [27] are applicable to the multivariate case. However, the model was only described without fitting or application, therefore we have not included these association structures here

#### Time-dependent associations

The standard joint model assumes that risk of an event at time *t* depends on the true value of the longitudinal profile for the same time point (Table 1, A1)—the so-called current value parameterization. The strength of the association is fully interpretable: exp(*α*_*k*_) is the hazard ratio for a unit increase in *μ*_*ik*_(*t*), at time *t*. An alternative current value parameterization is to replace the linear predictor term by the expected value of the longitudinal trajectory function at time *t*, *h*_*k*_^− 1^(*μ*_*ik*_(*t*)) (Table 1, A2), which is of importance for correctly modelling the functional form. The current value parameterization can be extended to incorporate additional structure, including interaction terms with the covariates (Table 1, A3), which might yield more realistic inferences as it is conceivable that different associations exist for different patient subgroups. In some cases one might posit that the risk depends not on the current value, but on a previous value, giving rise to the time-lagged values parameterization (Table 1, A4). Current value parameterizations have been used in model frameworks not compatible with (3). Chi and Ibrahim [42] adopted a novel bivariate time-to-event model whereby covariates—including a current values parameterization—are entered by a method corresponding to a canonical link in a Poisson generalized linear model. Song et al. [38] developed a model such that *W*_*i*_(*t*) = *α*^*T*^*ψ*(*t*, *b*_*i*_), for some vector function *ψ* (Table 1, A5). The estimation methodology assumed that *ψ*(*t*, *b*_*i*_) could be factorised into *ψ*(*t*, *b*_*i*_) = *ψ*(*t*)*b*_*i*_, which admits the current value parameterization as a special case, and leads to a number of extensions including interactions with time. In cases where *ψ*(*t*, *b*_*i*_) does not factorise, meaning that it is nonlinear in *t*, the authors propose using a linear approximation method.

Derivative terms allow one to incorporate not only the current value of the true longitudinal process, but also the rate of change, which intuitively might be associated with risk of the event. For example, two patients might have the same observed longitudinal outcome at time *t*, but one patient’s trajectory might be rising considerably more quickly than the other patient’s. *W*_*i*_(*t*) can therefore be augmented to include the current value plus the first *V* derivate terms (Table 1, A6), although this model is typically only used with the first order derivatives (*V* = 1), giving rise to the time-dependent slopes parameterization. The antithesis of the time-dependent slopes parameterization is the cumulative effects parameterization (Table 1, A7). Here, a summary of the entire history of the longitudinal process up to time *t* is included in the hazard model, *λ*_*i*_(*t*). This is contrary to other association structures that relate the hazard function only to features of the longitudinal model at a fixed time point.

#### Random effects parameterization

The above parameterizations often require numerical integration, which presents a computational challenge. Simpler time-independent associations can overcome this. The random effects parameterization (Table 1, A8), which only includes the time-independent random effects, is therefore frequently used in joint models. This parameterization has been used by a number of authors in various ways. In the simple case of a random-intercepts and random-slopes model for (2), the random effects represent the subject-specific deviation from the average intercept and slope fixed effect terms. Nevertheless, experts have echoed caution when attempting to use these models for inference, as complex longitudinal trajectory functions, such as those modelled by polynomials or splines, lead to non-interpretable association parameters [4, 15]. On the other hand, Jaffa et al. [50, 51] were specifically interested in the multivariate LMM slopes, so there was a clear a priori rationale for this association structure. Moreover, they noted that their model does not preclude inclusion of the random-intercepts, but demonstrated that specifying the marginal time-to-event (dropout) model is only required. Random effects parameterizations are sometimes used to refer to models where the hazard is associated with random plus fixed effect terms (Table 1, A9). For example, rather than model risk as dependent on *b*_*ik*_, one assumes it is dependent on ${b}_{ik} + {\tilde {\beta}}_k^{(1)}$, where ${\tilde {\beta}}_k^{(1)}$ is a subset of *β*_*k*_^(1)^ that correspond to the random effects terms in *b*_*ik*_. This model can be generalised to include functions of random coefficients.

#### Correlated random effects and frailty

An alternative approach to specifying an association structure is not to directly include random effects components of the longitudinal submodel in the time-to-event submodel, but rather to include separate random effects in each, and specify a joint distribution for the latent terms (Table 1, A10). In the simplest case, one can set *W*_*i*_(*t*) = *θ*_*i*_, and then jointly model (*b*_*i*_^*T*^, *θ*_*i*_)^*T*^. Rizopoulos and Ghosh [29] considered such a structure, assuming that the joint distribution was unknown, and used a Dirichlet prior to fit the model.

#### Correlated random effects and error

Pantazis et al. [40], Thiébaut et al. [41], and Pantazis and Touloumi [43] assumed a log-normal distributional for the event times, i.e. log(*T*_*i*_) = *X*_*i*_^(2)^*β*^(2)^ + *e*_*i*_, where the error terms *e*_*i*_ are assumed to follow a normal distribution with mean 0 and variance *σ*_*T*_^2^. The multivariate longitudinal data submodel and log-normal event time submodel were associated by assuming that (*b*_*i*_^*T*^, *e*_*i*_)^*T*^ are jointly distributed. When the random effects are multivariate normally distributed, this distribution is also multivariate normal. The covariance terms cov(*b*_*ik*_, *e*_*i*_) can then subsequently be used to quantify and test the strength of association.

#### Joint latent class models

An alternative approach to joint modelling is the use of joint latent class models (JLCMs). The assumption underlying JLCMs is that the population of subjects is heterogeneous, but consists of a number of homogeneous latent subgroups for which the subjects share the same mean longitudinal trajectories and hazard risk. A review of JLCMs is given by Proust-Lima et al. [33]. A class-specific *latent process mixed model*, which we refer to as the *latent variable model* to remain consistent with other similarly structured models, conditional on subject *i* being in class *m*, is then specified following a standard LMM: ${\mu}_i\left({t}_{ijk}\right)\Big|{}_{c_i=m}={X}_i^{(1)}\left({t}_{ijk}\right){\beta}^{(1)}+Z\left({t}_{ijk}\right){b}_{im}$. The fixed effects coefficients can also be made class-specific [57]. The observed multivariate longitudinal data are modelled using GLMMs or other suitable *measurement models* conditional on this latent variable: *h*_*k*_(*Y*_*ijk*_) = *μ*_*i*_(*t*_*ijk*_) + *α*_*ik*_ + *ε*_*ijk*_, for some suitable link functions *h*_*k*_(⋅). If *β*^(1)^ is forced to be class-specific, then one might introduce additional covariates with a global fixed effects coefficient vector into the measurement model [57]. There are two sets of random effects in this model: the subject-class effects in the latent process model, and the subject-outcome effects in the longitudinal data submodel. The time-to-event submodel might be modelled as a proportional hazards model, *λ*_*i*_(*t* | *c*_*i*_ = *m*) = *λ*_0*m*_(*t*)exp(*X*_*i*_^(2)^*β*_*m*_^(2)^). The class membership probabilities are modelled using multinomial regression models. As JLCMs do not require precise modelling of the association structure between the different submodels, with the association captured entirely by the latent classes, this means that they are not necessarily well suited for evaluating assumptions regarding the association between the two submodels; however, they are particularly useful for predictive modelling.

#### Other

Other MVJM approaches that fall outside of the ubiquitous shared random effects model framework or the emerging joint latent class model framework, lead to alternative association structures. Fieuws et al. [63] used a pattern-mixture model approach. Here, the dependency derived from the longitudinal submodels being fitted conditional on the failure times. Hu et al. [48] proposed a model that incorporates some function of the history of the longitudinal data, which reduces to the current value parameterization as a special case. Bartolucci and Farcomeni [61] proposed a discrete time event-history model with a mixed latent Markov model. A flexible association structure was obtained though the introduction of two discrete latent variables: a time-varying latent variable distributed according to a finite first-order latent Markov chain, and a time-constant latent variable.

### Estimation techniques

Historically, complete likelihood analysis was precluded by the inherently complex likelihood functions, necessitating so-called two-stage models [9, 10]. However, these models have been shown to lead to biased results [77]. A number of estimation approaches have been considered for MVJMs, building on the methodological developments in the univariate joint model literature [5, 78].

#### Frequentist model estimation

The expectation-maximisation (EM) algorithm [79] was the original estimation approach for joint-likelihood univariate joint models [5, 6], and therefore continues to be employed in a number of MVJM approaches [37, 40, 43, 61, 68]. At the M-step, maximization was routinely implemented using both closed-form estimators and the Newton-Raphson algorithm. Some noteworthy extensions included the one-step-late algorithm [37] and restricted iterative generalized least squares [40, 43]. The E-step was typically implemented using numerical integration, including Gaussian quadrature (e.g. [adaptive] Gauss-Hermite), although Monte Carlo integration [37], extensions of the forward-and-backwards recursion method [61], and exploitations of multivariate normal and truncated normal distributions [40, 43], were also implemented. Full maximum likelihood estimation can also be implemented directly by Newtonian-like approaches. These include the Marquardt algorithm [41, 44, 45, 57], Newton-Raphson algorithm [62], and robust variance-scoring algorithm [52]. Huang et al. [66] used automatic differentiation—a numerical technique for simultaneously evaluating a function and its derivatives—with a Newton-Raphson algorithm, which was purportedly faster than the EM algorithm.

For estimation methods based on likelihood maximization, evaluating an approximated inverse observation matrix at the maximum likelihood estimate is a standard approach [37, 41, 45, 52, 61, 62, 66] of calculating standard errors. Semiparametric time-to-event models have been noted to result in underestimation of parameter standard errors [80] in the univariate joint model framework, and can be unfeasible as the information matrix increases with sample size. One approach to overcome this is the bootstrap method, which has been adopted in MVJM approaches [46, 68]. Pantazis et al. [40] and Pantazis and Touloumi [43] estimated standard errors by refitting the model with multiply imputed data for censored survival times, which is quicker than conventional EM algorithm approaches.

#### Bayesian model estimation

The Bayesian approach has a number of advantages and has been previously exploited in the univariate joint modelling framework [78]. One such advantage is the ease of incorporating hierarchical data, as used by Luo and Wang [18] as part of a multicentre RCT joint model. Another advantage is the availability of Markov chain Monte Carlo (MCMC) sampling algorithms, which allow estimation from posterior probability density functions that are not analytically tractable, and which require complex multi-dimensional integration over the random effects. The Gibbs sampling algorithm has been the standard choice [17–19, 29, 39, 42, 47, 49, 53–56, 59, 60, 64, 65, 67, 70], applied in conjunction with the adaptive rejection algorithm, slice sampling algorithm, block sampling, and Metropolis-Hastings algorithm. The surge in Gibbs sampling can be partly explained by the use of the BUGS computer language [81], in particular WinBUGS and OpenBUGS, which reduces the need for complex analytical derivation. Deriving the likelihood function, however, is still challenging, and depending on the time-to-event model and latent association, numerical integration might be required. For example, Ibrahim et al. [19] used an approximation similar to that used by Tsiatis et al. [10] that is convenient so long as the response does not change over time too rapidly compared to the scheduled longitudinal data measurements. Brown et al. [39] on the other hand used a simple trapezoidal rule. Gaussian quadrature, such as the Gauss-Kronrod rule, has also been used [29, 56, 59, 60]. Liu and Li [47] compared the performance of Bayesian approaches to classical frequentist (maximum likelihood) approaches under different strengths of association, demonstrating superiority of the Bayesian methods with respect to bias, root-mean square error, and coverage. Another advantage to Bayesian modelling is the ability to incorporate prior knowledge. Tang and Tang [49] explored the sensitivity of results to prior distribution selection, showing that good prior knowledge led to marginally improved estimation. Uncertainty about posterior parameter estimates is readily calculated from the MCMC output without need for further complex calculations.

#### Other estimation approaches

Song et al. [38] extended the semiparametric conditional score estimator for the parameters in the hazard relationship, as proposed by Tsiatis and Davidian [14] in the univariate framework, which treats the random effects as nuisance parameters, and a set of estimating equations are deduced based on a derived sufficient statistic. Parameter standard errors were subsequently estimated using a sandwich matrix estimator. Li et al. [68] employed a non-trivial extension of a method proposed by Tsonka et al. [82] in order to estimate the model parameters and zero-mean random effects distribution using a modified vertex-exchange method algorithm in conjunction with an expectation-maximization algorithm. Hu et al. [48] circumvented the classical joint modelling framework by proposing a multiple imputation algorithm using either a fully conditional specification or MCMC approach, such that simple and transparent statistical approaches can be separately applied to the complete data. Rubin’s rule [83] could then be used to account for the additional uncertainty in standard errors from imputation.

Albert and Shih [46] proposed a novel two-stage regression calibration approach. In the first stage, conditional on each subject’s event time, complete longitudinal data were simulated for each subject using normal approximations. Multivariate longitudinal models were then estimated using the approach of Fieuws and Verbeke [84], which fits all bivariate models and averages over the duplicate parameter estimates. This method is advantageous as it enables one to consider high-dimensional data, which would otherwise present numerical challenges or be computationally infeasible. Following this, an estimator was proposed for the subject-specific random effects, allowing for estimates of the mean longitudinal trajectories at each discrete time point for each subject to be obtained. In the second stage, a regression-calibration approach was then used to estimate the discrete time-to-event model parameters. The resulting parameter estimates were averaged over repeated simulations of the model-fitting algorithm. Standard errors were estimated using the bootstrap method.

Fieuws et al. [63] adopted a pattern-mixture model estimation approach, whereby multivariate longitudinal models are estimated—separately for those who experience and those who do not experience the event—again using the proposed approach of Fieuws and Verbeke [84] described above. Bayes’ rule was then used to estimate the failure time distribution conditional on the longitudinal profiles, with a non-parametric survival distribution acting as the prior distribution.

### Software

The adoption of joint models has been slow [15]. Among the many reasons for this includes the historically limited availability of software specific to joint models. Recently packages for mainstream statistical software, including R (R Foundation for Statistical Computing, Vienna, Austria) [24, 27], SAS (SAS Institute, Cary, NC) [26], Stata (StataCorp LP, College Station, TX) [22], and WinBUGS (MRC Biostatistics Unit, Cambridge, UK) [23], have allowed researchers to exploit joint modelling. However, these have been limited to univariate data. Many articles describing developments or applications of joint models involving multivariate longitudinal data have reported some details about the software used to fit the joint models.

#### R

Brown et al. [39] compiled their flexible B-spline model for multiple longitudinal biomarkers and time-to-event outcome (with current value association parameterization) into an R package, sjmsoft, available from the author’s website: http://faculty.washington.edu/elizab/software.html [Accessed: 25 January 2016]. Battes et al. [21] used the R package JM [27], which fits univariate joint models, by reducing the multivariate longitudinal data to a univariate outcome through ad hoc techniques. R has also been used as an interface to execute JAGS [85] and WinBUGS/OpenBUGS programs [47, 53, 56, 65]. Andrinopoulou et al. [59] implemented their model using two separate software packages, one of which was R, with the code available from the authors upon request. Tang et al. [60] reported their Bayesian models were fitted using R and Matlab (The MathWorks Inc., Natick, MA), with code available from the authors upon request. Albert and Shih [46] fitted the bivariate longitudinal models using code presented elsewhere [86]. Bartolucci and Farcomeni [61] published R code as a supplemental file for their event-history extension Markov model, which depends on compiled Fortran routines.

#### BUGS & JAGS

Bayesian MVJMs have been coded in the BUGS language and fitted using WinBUGS and OpenBUGS [18, 29, 53–55, 59, 64, 65]—software that implements MCMC sampling. JAGS has also been used as alternative [47, 56], which shares a similar syntax to BUGS models. The flexibility of the software has permitted countless association structures, submodels, and data types. Hatfield et al. [53] also noted that they extended the WinBUGS platform to include a ‘zero-augmented beta’ distribution using code written in Pascal. In the majority of publications, the model script has been made available in an appendix [18, 29, 55, 59, 64] or is available on request from the authors [56, 65]. However, it was generally application specific; thus requiring adaptation for application with other datasets.

#### Stata

It has recently been announced that the next version release of the Stata package stjm [22, 87] will allow for multivariate longitudinal outcomes. The current version implements maximum likelihood estimation of univariate joint models with a number of different parametric time-to-event models. In addition, stjm can jointly model different outcome types, different association structures, and different random effects covariance matrix structures, alongside extensive optimization control settings, thus giving the user immense flexibility. A number of post-estimation options are available, including residual calculation and prediction. Pantazis et al. [40] also report that a Stata program is available (on request from the authors) for fitting their bivariate MVJM.

#### Fortran

Fortran has been used in several MVJM studies [19, 44, 57, 61]. Of particular interest is the HETMIXSURV (version 2) program available from: http://www.isped.u-bordeaux.fr/BIOSTAT [Accessed: 11 April 2006]. This Fortran 90 parallel program implements maximum likelihood estimation of the multivariate JLCM proposed by Proust-Lima et al. [57], in addition to other related models, which permits different outcome types and submodels. The R package lcmm [28] has similar capabilities, but does not currently permit multivariate longitudinal data in the JLCM framework. Dantan et al. [44] also fitted a JLCM using Fortran 90, and note the code is available on request. Ibrahim et al. [19] also report code is available for fitting their MVJM and multivariate *L*-statistic from the authors’ website.

#### Other

SAS has been implemented in the MVJM literature, with direct reference to the procedure NLMIXED [20, 50, 51, 63], but without making the code available. Song et al. implemented their conditional score method using C++ [38], and Li et al. [68] fitted their bivariate ordinal model with competing risks data using C. In both cases, the code is available from the respective authors upon request. Matlab was used by Lin et al. [37] to run the one-step-ahead EM algorithm (code not provided), and by Tang et al. [60] to implement an MCMC algorithm for a Bayesian MVJM (code available on request from the authors). Huang et al. [66] developed an S-Plus (Insightful Corporation, Seattle, WA) library model, AD09, to implement the automatic differential method that enables direct maximization of the MVJM likelihood function. In addition to a Stata program, Pantazis et al. also reported that MLn (Centre for Multilevel Modelling, University of Bristol, UK) macros are available from the authors for fitting their bivariate MVJM.

### Clinical examples

Most methodological developments in the MVJM framework have been motivated by real-world clinical studies. Applications have been in the disease areas of HIV/AIDS [16, 38–41, 43, 52], lung disease [65, 68], cancer [19, 37, 42, 49, 53, 60, 70], cardiovascular disease [21, 56, 59, 67], neurodegenerative disease [18, 45, 54, 55, 57], renal disease [17, 29, 48, 50, 51, 63], hepatic disease [46, 61], mental health [58, 66], and cognitive studies [20, 44]. We illustrate three examples below.

#### Parkinson’s disease drug trial

Parkinson’s disease is a chronic progressive neurodegenerative disorder. Luo and Wang [18] reported data from a clinical trial of 800 patients randomized in a 2x2 factorial design to receive double-placebo, tocopherol, deprenyl, or tocopherol with deprenyl. The latter two groups formed the treatment group, whilst the former two defined the placebo group. To investigate the effect of tocopherol in slowing the progression of Parkinson’s disease, 3 longitudinal outcomes were recorded that describe progression: 1 continuous and 2 ordinal measuring different facets of the disease. A substantial number of patients (376/800) failed to complete the measurement schedule (10 measurements over a 24-month follow-up period), due to deterioration and were treated with levodopa therapy. The time to initiation of levodopa therapy was the study endpoint. It was shown that patients with shorter follow-up had worse progression measurements on average; therefore, a multivariate joint model was required to account for the informative dropout. For the multivariate longitudinal data, a multilevel item-response theory model was used, with centre effects included to account for the cluster-trial design. A piecewise constant proportional hazards regression model was used for the time-to-event data, with a shared (centre-specific and subject-specific) random effects association structure (Table 1, A8). Based on multiple model comparison methods, a random effects parameterization without centre-level effects was optimal. This model indicated a non-significant effect of tocopherol on disease progression rate and on the hazard of levodopa therapy initiation when compared to placebo. However, the association parameters were strongly significant, indicating that patients with worse disease severity and faster disease progression had an increased hazard of levodopa therapy initiation. This data was also previously analysed by Luo [55] and He and Luo [54] without consideration of the centre effects, and in the former case under different parametric time-to-event models.

#### Heart valve replacement observational study

Aortic valve replacement is a common cardiac surgery procedure, often required to treat aortic stenosis. Andrinopoulou et al. [59] analysed data from a cohort on 283 patients who survived aortic valve or root replacement with an allograft valve. During the routine clinical follow-up appointments, two echocardiographic measurements were recorded: valve gradient (continuous) and regurgitation (ordinal). Physiologically, increased gradient or regurgitation indicates deterioration of valve performance, which could lead to either death or necessitate reoperation—both events of interest. To investigate the effects of these parameters on the hazard of adverse events, a MVJM was proposed for the bivariate longitudinal data and a competing risks model was assumed for the event times. A LMM with B-spline functional form was used to model the non-linear gradient trajectory, and a continuation ratio mixed effects model for the regurgitation data. A simple LMM for aortic gradient was considered, but resulted in an inferior model fit. A cause-specific event-time model with piecewise constant baseline hazards was used to model the competing risks data, with a random effects (+ fixed effects) association structure (Table 1, A9). The association between the longitudinal measurements and events are presented in the form of graphical plots to facilitate interpretation, as the B-spline parameters are not straightforwardly interpretable. Andrinopoulou et al. [56] reanalysed this data with the purpose of calculating dynamic predictions. Furthermore, they extended the model to consider several different association structures, with subsequent predictions combined using Bayesian model averaging in order to account for model structure uncertainty.

#### Cancer drug trial

Lin et al. [37] reanalysed data from a placebo-controlled randomized trial to establish whether supplemental beta-carotene reduces recurrence of the primary tumours in patients cured from a recent early-stage head and neck cancer. The primary endpoint was all-cause mortality, with 63 subjects dying during follow-up. During the trial, blood samples were taken from 264 patients at baseline, 3-months, 1-year, and annually thereafter until 5-years total follow-up was attained. Two continuous biomarkers—both plasma nutrient concentrations—were of interest: lycopene and lutein + zeaxanthin. Previous univariate studies had demonstrated that increased biomarker concentrations were associated with reduced mortality. Interest here was specifically on whether both biomarkers were simultaneously associated with mortality. To investigate this, a MVJM was developed with LMMs used to model the biomarkers, and a Cox PH model to model the time to death, with association modelled using an interaction parameterization (Table 1, A3). Additionally, a gamma-distributed subject-level frailty term was included to capture over-dispersion. It was found that the effect of lutein + zeaxanthin was diminished when both biomarkers were included, and moreover the sign was opposite to that of the univariate joint model. By exploring the correlation between the biomarkers, the authors suggested that the effect of lutein + zeaxanthin on all-cause mortality appeared to be mediated only through the association with lycopene. Lin et al. also demonstrated the necessity of joint modelling by fitting (1) a separate event time model with baseline biomarker concentrations; (2) a time-varying covariate Cox model (equivalent to a last observation carried-forward model); and (3) a two-stage joint model, and juxtaposing each to the fitted MVJM.

## Discussion

The case for use of joint models has been made in the univariate data framework [3]. Similarly in the MVJM literature, several studies have demonstrated the potential bias from ignoring the correlation between the outcomes by comparing joint models to separately fitted submodels in simulation analyses [18, 40, 42, 47, 54, 55]. With an increased focus on personalised medicine, the need to implement models that account for multiple longitudinal outcomes is necessary. Despite this, joint models have predominantly focused on univariate data. A consequence of this has been researchers fitting multiple separate univariate joint models [88], which is inefficient and can affect inferences [37]. Here we have presented an overview of MVJM literature. The models developed in the literature showcase broad classes of longitudinal and time-to-event data and countless association structures, with bespoke models often developed to meet the demands of the clinical data at hand. A small number of models have even considered simultaneously multivariate longitudinal data and multivariate time-to-event joint models [17, 42, 48, 49, 56, 59, 60, 66, 68, 70].

One advantage of joint models is that they might improve power and efficiency over separate fitted models [3]. Nonetheless, few applications of joint models to clinical trial data have derived from a planned joint modelling design. For joint models to become embedded in clinical research, researchers will need guidance on study design with reference to the different models and estimation proposals available. However, issues such as statistical power have attracted little attention in the univariate joint model literature [11, 89]. Furthermore, developments in the field of joint modelling—particularly MVJMs—have primarily focused on modelling and estimation. Practitioners will naturally require diagnostic tools to evaluate model fits and compare models. Articles considering MVJMs have utilised many different model comparison methods, goodness-of-fit tests, and diagnostics. In some cases, these statistical methods have been developed specifically for the multivariate data joint modelling framework. We illustrate some of the approaches adopted in the literature in Additional file 1: Table S1.

The extension from univariate data modelling to multivariate data modelling is, in principle, straightforward [4]. The main barrier to applied researchers is the lack of readily available software that can implement multivariate joint model estimation approaches; this is despite an emergence of software relevant to univariate joint models. The main limitation to statisticians is the inherent computational challenge that arises from the increasing dimensionality of the random effects component. Despite many multivariate models being presented in full generality, computational power, limits in numerical estimation, (effective) sample size, and clinical data availability will, unavoidably in practice, preclude analyses involving large dimensions. Of the MVJM methodologies encountered to-date, bivariate data is the most commonly encountered during application [19, 20, 37–41, 43, 48, 50, 53, 56, 57, 59, 61, 64, 68], followed by trivariate data [18, 21, 29, 44–47, 51, 58, 66]. A few articles have considered 4 longitudinal outcomes [17, 42, 49, 54, 55, 60, 63, 70]. Jaffa et al. [51] explored converge properties of their MVJM for up to 10 longitudinal outcomes, and reported good performance. It was also noted that the model should straightforwardly extended to higher dimensions. Of all the studies involving multiple event types or competing risks, all were limited to the bivariate case/two competing risk events, with the exception of one study [17], which considered 9 separate recurrent events along with 4 longitudinal biomarkers. However, in the latter case it was reported that a single joint model could not actually be fitted due to computational limitations, with the authors opting instead to fit multiple pairwise models.

The flexibility of a model to be applied to a given dataset will depend on the ability to include complex functional forms and covariate adjustment options within the submodels. Complex functional forms, whilst perhaps better capturing non-linear trajectories in the longitudinal submodel that may be observed in biological data, increase computational requirements to fit as the dimensions of the random effects increases. Notwithstanding these issues, some have developed models which includes complex smoothing functions within the multivariate framework, including B-splines [39, 59], natural cubic splines [29, 56, 62], fractional polynomials [62], P-splines [49], thin-plate splines [17], I-splines [57], and parametric non-linear functions [43]. Albert and Shih [46] also noted that their approach could be extended to nonlinear models with an additional approximation. The mechanistic model proposed by Guedj et al. [52] is able to model complex dynamics of biomarkers by virtue of the complex system of differential equations specified, which captures knowledge of actual biological processes.

## Conclusions and Recommendations 

Gould et al. [15] concluded that it was going to be a challenge to encourage stakeholders to adopt joint models. This challenge is further exacerbated when multivariate longitudinal data are incorporated. However, as demonstrated in this review there is a solid methodological foundation to implement these methods. We have also observed the early phase of infiltration into the non-statistical biomedical literature [16, 20, 21], which highlights the potential of the MVJM framework. For MVJMs to be integrated into the applied biostatistician’s tool belt, further developments are required, including:

- Development of statistical software capable of fitting of MVJMs. Univariate joint models have seen a surge in software development, which includes wider integration with multivariate event-time data; however, lack of software for multivariate longitudinal data will preclude their use by the vast majority of applied statisticians.
- Reporting of the maximum dimension limits for multiple longitudinal outcomes. Whilst many models are presented in full generality, computational limitations will preclude large numbers of random effects, and therefore large numbers of biomarkers. Currently, little is understood about this with the exception of short commentaries by Jaffa et al. [51] and Musoro et al. [17].
- Guidance on the underlying model types, distributional assumptions, and choice of association structure. Despite methodological developments, a coherent and flexible modelling framework that encapsulates the general multivariate joint model is lacking, which precludes penetration into the biomedical arena due to a lack of understanding.
- Development of model diagnostics and selection devices compatible with the MVJM framework. A number of useful ancillary statistical tools have been recently presented, however clarity and demonstration of their suitability, as well as integration into the aforementioned software developments, are required.
- Study design guidance, including but not limited to: sample size and power calculations; selection of appropriate latent association structures; applicability of models according to different intra- and inter-patient measurement protocols, and outcome types; surrogacy; and dynamic prediction.
- Further research into MJVMs with multivariate time-to-event submodels, including competing risks, recurrent events, multiples events, and multistate data.

## Additional file

Additional file 1: Table S1. Goodness-of-fit tests, model diagnostics, comparison instruments, and other model assessment tools. (DOCX 162 kb)

## Abbreviations

GFR: Glomerular filtration rate
GLMM: Generalised linear mixed model
JLCM: Joint latent class model
LMM: Linear mixed model
MCMC: Markov chain Monte Carlo
MVJM: Multivariate joint model
PH: Proportional hazards
