# Supplementary material for: Joint modelling of time-to-event and multivariate longitudinal outcomes: recent developments and issues
Source: BMC Med Res Methodol. 2016 Sep 7;16(1):117. doi: 10.1186/s12874-016-0212-5 (PMC5015261; doi:10.1186/s12874-016-0212-5)
Supplement: Additional file 1: Table S1. — Goodness-of-fit tests, model diagnostics, comparison instruments, and other model assessment tools. (DOCX 162 kb) [file 12874_2016_212_MOESM1_ESM.docx]

**Table S1.** Goodness-of-fit tests, model diagnostics, comparison instruments, and other model assessment tools.

|  | **Description** | **Used** | **Studies** |
| --- | --- | --- | --- |
| **Information criteria** |  |  |  |
| - Akaike information criterion (AIC) - Schwartz information criterion (BIC) - Deviance information criterion (DIC) - Hannan-Quinn information criterion (HQIC) | A measure of model fit that trades-off goodness-of-fit against model complexity. | Model selection of:   - different trajectory functions - number of spline basis functions - covariance structures - adjustment covariates - number of latent classes - latent association structure - model distributions (for both time-to-event and longitudinal models) | [17, 18, 21, 29, 39, 42, 45, 49, 52–57, 59, 61, 64, 65] |
| **Other model comparison statistics** |  |  |  |
| LRT | Statistical test used to compare the goodness of fit of two models. | Assess whether covariates are non-zero, including latent association parameters. | [20, 37, 38, 40, 44, 45, 63] |
| Bayes factors | Bayesian alternative to *P*-values for testing hypotheses and for quantifying the degree to which observed data support or conflict with a hypothesis. | Model comparison with reduced models. | [18, 54] |
| Log pseudo-marginal likelihood statistic (LPML) | Combines information on conditional predictive ordinates (CPOs) for model selection, where the CPO is a Bayesian diagnostic that detects surprising observations, which can also be used individually | Model selection of:   - number of knot for B-spline basis - time-to-event model - latent association structure | [39, 53, 56, 64] |
| Root mean squared prediction error (RMSPE) | Measures the model’s ability to predict outcomes not used in model fitting by holding out a fixed percentage of subjects from the model fit sample | Model selection of random effects correlation structure. | [53] |
| **Residuals** |  |  |  |
| Martingale residuals | Identification of excess events in the model to evaluate functional form adequacy in the hazard function. | Model fit assessment. | [62] |
| Cox-Snell residuals | Check model fit and assess the appropriateness of the model. | Model fit assessment. | [29] |
| Longitudinal submodel residuals  (marginal, Studentized / standardized conditional) | Assess model assumptions in the longitudinal submodel. | Assess:   - homoscedasticity assumption - outlier detection - linearity assumption | [38, 40, 62, 64] |
| Conditional residuals | Testing the conditional independence assumption, which assesses whether conditional on the latent classes if there is any residual dependency between the longitudinal and time-to-event submodels captured through a random effects parameterization. | Assess the hypothesis of conditional independence in the joint latent class model. | [45] |
| Score test | A formal statistical test based on comparing the mean conditional residuals between censored and uncensored subjects. | Assess the hypothesis of conditional independence in the joint latent class model. | [45, 57] |
| **Novel diagnostics and statistics** |  |  |  |
| Measures of relative benefits of multiple longitudinal biomarkers as opposed to a single one | Two complementary measures of relative benefit:   1. a measure of gain in precision for the ratio of the cumulative hazards at maximum follow-up 2. a measure of the estimated additional direct effect of the treatment not mediated through the markers | Assess the relative benefits of multiple surrogates versus a single one. | [58] |
| Multivariate *L*-measure | Bayesian model selection statistics. | Comparing models with:   - different longitudinal trajectory functions - number of intervals and knot positions for piecewise constant baseline hazard function - error distribution correlation - random effects distribution correlation structure | [19] |
| Bayesian case-deletion influence diagnostic | Assess the effect of subjects on parameters estimated based on the *φ*-divergence function, which includes the Kullback–Leibler divergence and *L_1_*-divergence as particular cases. | Detect the potential influential observations. | [60] |
| Bayesian local influence measures | Characterizes the direction and largest degree of model perturbations with respect to three classes of perturbation models. These models explored the effects of perturbations to:   1. the data points (longitudinal and time-to-event) to detect one, or a few, subjects with influential profiles 2. the shared random-effects distribution to detect either influential survival times, whose occurrence is inconsistent with the corresponding longitudinal profile, or to detect influential random effects 3. the prior distribution to assess sensitivity of posterior quantities to prior knowledge | - Explored on different model comparison statistics, including the *φ*-divergence function, Bayes factor, and posterior mean distance function. - The ability of the influence measures to detect model perturbations was demonstrated by simulation analysis. - Assess the effect of minor perturbations to within-subject measurement error and random effects. | [49, 70] |
| Calculation of the posterior classification tables | Characterization of the classification of the subjects using the posteriorly calculated class-membership probabilities. | To assess the goodness-of-fit and discriminatory ability of JLCMs. | [57] |
